# Optimization of bacteriocin production by *Lactobacillus rhamnosus* CW40: exploring its therapeutic and antibacterial scope

**DOI:** 10.3389/fmedt.2025.1663924

**Published:** 2025-09-12

**Authors:** Preeti Upadhyay, Abhishek Kumar Verma, Harshada Joshi

**Affiliations:** ^1^ Department of Biotechnology, Mohanlal Sukhadia University, Udaipur, Rajasthan, India; ^2^ Department of Zoology, Jai Minesh Adivasi University, Kota, Rajasthan, India

**Keywords:** LAB, cow milk, bacteriocin, probiotic, gel filtration chromatography, *Lactobacillus rhamnosus* CW40, microbial therapeutic

## Abstract

Bacteriocins are ribosomally produced, proteinaceous antimicrobial biomolecules with versatile functions and are considered potential next-generation therapeutics. They are secreted by a few groups of lactic acid bacteria (LAB) that possess the ability to combat spoilage and foodborne pathogens. Given these properties, bacteriocins have generated significant interest for their potential use as natural food preservatives. This study aimed to isolate and characterize bacteriocin-producing LAB with potent antimicrobial properties and evaluate their potential use as natural food preservatives and alternative therapeutics. A total of 47 morphologically distinct LAB isolates were screened for antibacterial activity against foodborne pathogens. The isolate exhibiting the strongest antimicrobial activity, designated CW40, was selected for further study. The bacteriocin was purified from the culture supernatant using gel filtration chromatography. The isolate was identified by 16S rDNA sequencing, and optimal conditions for bacteriocin production were determined. The molecular weight of the bacteriocin was estimated, and its antimicrobial spectrum, enzyme sensitivity, bile salt tolerance, and antibiotic resistance profile were assessed. Isolate CW40 produced 8 kDa (MW) of bacteriocin from the purified supernatant of its grown culture and was identified as *Lactobacillus rhamnosus* through 16S rDNA sequencing. The bacteriocin production of *L. rhamnosus* was optimized, with maximum yield observed at 37℃ with pH 7. The bacteriocin exhibited strong antimicrobial activity against *Bacillus subtilis*, *Bacillus cereus*, and *Escherichia coli*. Protease treatment eliminated antimicrobial activity, confirming the proteinaceous nature of the bacteriocin. Maximum bacteriocin activity at 4,098 AU/mL was observed against *E. coli*. The strain tolerated up to 0.3% (w/v) ox gall and demonstrated a broad antibiotic resistance. The results highlight *Lactobacillus rhamnosus* CW40 as a promising source of bacteriocins with potent antimicrobial properties. These findings support the potential application of CW40-derived bacteriocins as natural biopreservatives and adjunct therapeutic agents in combating foodborne pathogens and antibiotic-resistant infections.

## 1 Introduction

Probiotic bacteria confer their beneficial effects via mechanisms including competitive exclusion of pathogens, synthesis of antimicrobial compounds, improvement of gut barrier function, and modulation of immune responses. Milk and dairy products can serve as an excellent source for delivering probiotic bacteria. Cow milk provides a nutrient-rich environment, abundant in lactose and casein, that supports the growth and metabolic activities of lactic acid bacteria (LAB), thereby enhancing the likelihood of isolating potent bacteriocin-producing strains. High amounts of LAB, which are beneficial bacteria found in cow and goat milks, make them important for research on materials with potential public health benefits and applications in the dairy industry (1). Furthermore, probiotic bacterial vitality can increase after storage, presumably due to the lactose content in cow milk, which enhances bacterial survival. According to Asha et al. (2), lactic acid bacteria, specifically *Lactobacillus* abundant in cow milk, have shown significant broad-spectrum antibacterial activity against multidrug-resistant bacteria, especially through the expression of plantaricin (pln EF)-coding genes. By producing different substances during the fermentation of lactic acids, such as lactic acid, acetic acid, bacteriocins, diacetyl, and hydrogen peroxide, LAB exhibit antagonistic action against pathogens and food-spoiling microbes (3, 4). Bacteriocins are a diverse and large group of antimicrobial peptides that vary in physicochemical properties, molecular size, modes of action, antibacterial spectra, and source organisms (5). They are synthesized by all genera of lactic acid bacteria. Bacteriocins produced by LAB primarily exert their antimicrobial effect by forming pores in the bacterial cell surface and interfering with the proton motive force. Different kinds of bacteriocin, specifically Class I (lantibiotic bacteriocin), can have a binary mechanism of action (6). By adhering to the lipid II molecule, which is responsible for transporting peptidoglycan components from the cytoplasm, these bacteriocins can obstruct the proper formation of the cell wall. In addition, lipid II acts as a docking molecule to start cell membrane insertion, which causes pore formation and eventually leads to cell death. Class II peptides use their amphiphilic helical shape to penetrate the target cell membrane, causing depolarization and apoptosis. Class III peptides act directly on Gram-positive cell walls, causing lysis and death. Additionally, bacteriocin can function as a signal peptide to control the host immune system. According to Qiao et al. (7), serum immune components and intestinal flora of normal mice underwent considerable modifications after administration of bacteriocin-producing *Pediococcus acidilactici* strains. Due to their remarkable antibacterial activities, bacteriocins have been widely accepted as a viable natural antibacterial ingredient for food biopreservation. For instance, bacteriocins produced by LAB strains *Lactobacillus paracasei* LS-6, *Lactobacillus plantarum* CGMCC 1.12934, *L. rhamnosus* CJNU 0519, and *Lactobacillus plantarum* LF-8 have demonstrated strong inhibitory effects against foodborne *Staphylococcus aureus* (8–11). The Food and Agriculture Organization (FAO) defined probiotic bacteria as “live microorganisms that, when administered in suitable proportions, impart a health benefit on the host” (12). Among these, lactobacilli are well-known probiotic microbes that have been shown to generate metabolic products essential for regulating the growth of undesirable microflora in the gut. These bacteria are the most common inhabitants of the gastrointestinal tract (GI) in animals and humans. Lactobacilli have important qualities such as fighting off harmful bacteria, bile tolerance, and antibiotic resistance, which help them survive and persist in the gastrointestinal tract. One of the most popular probiotics in the food industry, *Lactobacillus rhamnosus*, has been shown to markedly reduce pathogen cell viability and substantially prevent biofilm formation (13). Rhamnocin is a bacteriocin produced by *Lactobacillus rhamnosus*, a probiotic lactic acid bacterium commonly found in the human gastrointestinal tract. Various Gram-positive bacteria, including foodborne pathogens such as *Staphylococcus aureus* and *Listeria monocytogenes*, are inhibited by this antimicrobial peptide (14, 15). Rhamnocin disrupts the target cell membrane, causing cell lysis, and is considered a promising natural preservative for food safety applications. This study aimed to (1) isolate and screen LAB from cow milk for bacteriocin production; (2) identify and characterize the most potent strain; and (3) optimize conditions for bacteriocin production and evaluate its antimicrobial and probiotic properties.

## 2 Materials and methods

### 2.1 Sampling, isolation, and purification of lactic acid bacteria

Cow milk samples were collected from a local dairy in the Indian state of Rajasthan, specifically in Udaipur. Lactobacilli were isolated using the pour plate method with de Man, Rogosa, and Sharpe (MRS) agar as the selective medium (16). The plates were incubated at 37°C for 48 h. After being recovered from the plates, the isolated colonies were transferred into a sterile MRS broth medium. These culture tubes that had been inoculated were kept at 37°C for 24 h. Following the observation of turbidity, the isolates were purified by successive streaking.

### 2.2 Antibacterial activity

The well diffusion method, as described by Ogunbanwo et al. (17), was used to assess the antagonistic activity against three bacterial strains: two Gram-positive, i.e., *Bacillus subtilis* (MTCC 121) and *Bacillus cereus* (MTCC 430), and one Gram-negative, *Escherichia coli* (MTCC 443). Antibacterial activity was measured using three preparations: cell-free supernatant without neutralization, cell-free supernatant neutralized with 1 N NaOH, and enzyme-treated neutralized cell-free supernatant. To ascertain the contribution of bacteriocin to the antibacterial activity, an enzyme treatment (protease-20 mg/mL) was conducted. Equal amounts of the enzyme and neutralized supernatant were mixed and incubated at 37°C for 2–3 h. The laterally extended zone of inhibition was measured using a scale. A clean zone of at least 1 mm was considered indicative of positive inhibition. Since the bacteriocins produced by *Pediococcus* spp. are categorized as class IIa bacteriocins and exhibit significant antibacterial activity, *Pediococcus acidilactici* LB42 was selected as the control.

### 2.3 Characterization of the isolate

The isolates were characterized based on cultural, morphological, biochemical, and molecular analyses. Cultural characterization was based on colony morphology, and Gram staining was used to examine the morphology. Biochemical characterization was based on the following: catalase reaction, growth on MRS agar supplemented with bromocresol purple (BCP), nitrate reduction, arginine hydrolysis, esculin hydrolysis, hydrogen cyanide (HCN) production, growth at different temperatures, and carbohydrate fermentation patterns using maltose, mannose, galactose, trehalose, and rhamnose.

Molecular characterization of the isolate was based on PCR-based product enhancement, amplified product sequencing, and genomic DNA extraction. Pospiech and Neumann's technique was used to isolate the genomic DNA (18). Based on a variable loop of 16S rDNA sequence designed by Klijn et al. (19), the isolate was exposed to PCR using semi-universal *Lactobacillus* genus-specific primers Lb1 (5′-AGAGTTTGATCATGGCTCAG-3′) and Lb2 (5′-CGGTATTAGCATCTGTTTCC-3′). The amplified products were sent to SciGenome Labs Pvt Ltd in Kochi, India, for sequencing. After BLAST analysis, the partly sequenced data were submitted to the EMBL-EBI database.

### 2.4 Production of bacteriocin

Bacteriocin activity was measured using a dilution method previously described (20) with slight modifications. The isolated bacteria were grown in MRS broth (10 mL) with 10% inoculum (10^8^ CFU/mL) from an overnight culture, and these cultures were incubated at 37°C. After centrifuging the samples at 8,000* × g* for 15 min at 4°C, the supernatant was filtered using a Millipore 0.22 μm Millex-GV filter.

In a 96-well microtiter plate, the supernatant was serially diluted twice in 125 µl of nutrient broth to measure the bacteriocin activity. As an inoculant, 50 µl of a 100-fold diluted overnight culture of the indicator strain was applied to each well. The microtiter plate was then incubated at 37°C for 16 h. Bacteriocin activity was calculated using the following formula and represented as an arbitrary unit per milliliter (AU/mL):

$$\mathrm{AU}/\mathrm{mL}=\frac{1,000}{125}\times \frac{1}{\mathrm{HD}}.$$

HD stands for the maximum dilution at which the indicator strain cannot grow. Using a spectrophotometer, optical density (OD) at 600 nm was measured to calculate the rate of bacterial growth.

### 2.5 Extraction, purification, and molecular weight determination

The isolated bacteria were grown in 1,000 mL of MRS broth with 10% inoculum (10^8^ CFU/mL) of overnight culture, and these cultures were incubated at 150 rpm for 48 h at 37°C. The entire broth was centrifuged at 1,000 × *g* for 15 min at 4°C after incubation, and the cell-free supernatant was then used as crude bacteriocin.

The cell-free culture supernatant (crude bacteriocin) was mixed with 70% ammonium sulfate to precipitate the proteins. The pellet was recovered after centrifugation at 1,000 × *g* for 30 min at 4°C. The sodium phosphate buffer (0.1 M, pH 7.0) was used to dissolve the pellet. Filtration was performed using the Zeba column; supernatant was filled in the Zeba column and centrifuged at 4,000 × *g* at 4°C. These protein samples obtained after desalting were analyzed for their antibacterial activity. The samples with the highest antibacterial activity were pooled and concentrated in a lyophilizer. Further protein purification was performed using gel filtration chromatography for the final polishing of protein using the Superdex 75 10/300 GL (Tricorn™ high-performance) column. A column was loaded with concentrated protein samples, and the flow rate was set to 0.4 mL/min. Fractions were collected and pooled after peaks had been achieved. Using the previously described agar well assay method, antibacterial activity was assessed for the fractions obtained at peaks to verify the existence of bacteriocin protein against indicator microorganisms.

Following Laemmli's method, 15% sodium dodecyl sulfate–polyacrylamide gel electrophoresis (SDS–PAGE) was used to determine the molecular weight of the isolated bacteriocin (21). After electrophoresis, the gel was stained with Coomassie Brilliant Blue R-250 and destained by washing it overnight in a 5:5:1 v/v solution of acetic acid, methyl alcohol, and water. The standard marker was the low-range molecular weight marker (Genei, India).

### 2.6 Effect of various factors on bacteriocin production by lactobacilli

The production of bacteriocin by the selected isolate was optimized by choosing one parameter at a time, including incubation temperature, initial pH, and salinity conditions. The experiment was performed in duplicate sets, and readings were taken using a spectrophotometer with a microtiter plate at 600 nm.

#### 2.6.1 Temperature

The effects of three temperatures (30°C, 37°C, and 45°C) on bacteriocin production were investigated for 24 h using MRS broth with an initial pH of 6.8 and without agitation. Bacteriocin production (AU/mL) was computed using the method outlined in the preceding section.

#### 2.6.2 pH

The effect of the initial medium pH on bacteriocin production was investigated by incubating the cultures at 37°C for 24 h. After adjusting the volume of 100 mL MRS broth to pH 4.0, 7.0, and 9.0 using either 1 M NaOH or 6 M HCl, the medium was autoclaved. Bacteriocin production (AU/mL) was computed using the method outlined in the preceding section.

#### 2.6.3 Salt concentration

A 100 mL amount of MRS broth with an initial pH of 7.0 was mixed with NaCl at various concentrations of 1%, 2%, and 3% before being autoclaved. Bacteriocin production (AU/mL) was computed using the method outlined in the preceding section.

### 2.7 Bile tolerance

The bile tolerance of the isolate was assessed using the method previously described (22). Ox gall supplied in MRS agar medium at varying concentrations (0.1%, 0.2%, 0.3%, 0.4%, and 0.5%) was employed. After streaking the medium, it was incubated at 37°C for 48 h.

### 2.8 Antibiotic resistance

The antibiotic resistance of lactobacilli was assessed using the disc diffusion method by Bauer et al. (23). Antibiotic discs (HiMedia) that were utilized included penicillin (10 units/disc), rifampicin (30 μg/disc), amikacin (10 μg/disc), cefixime (5 μg/disc), vancomycin (30 μg/disc), gentamycin (30 μg/disc), ampicillin (10 μg/disc), tetracycline (30 μg/disc), kanamycin (30 μg/disc), streptomycin (25 μg/disc), and trimethoprim (5 μg/disc). Following 24 h of incubation at 37°C, a scale was used to measure the inhibitory zone's diameter. This investigation was conducted in triplicate, and average values were computed. The data are displayed as mean ± SD.

### 2.9 Statistical analyses

All the observations recorded were subjected to the statistical analysis, viz., standard deviation (SD), using Microsoft Excel 2003. The significant differences among variable treatments were determined by the analysis performed in the JMP software (24) version 11 using the Tukey–Kramer honestly significant difference (HSD) test at *p* = 0.05.

## 3 Results

### 3.1 Sampling, isolation, and purification of isolates

Four cow milk samples were collected from the different dairies in Udaipur. On MRS agar, 47 isolates were recovered using the pour plate method. Subculturing on MRS agar resulted in the purification of each isolate.

### 3.2 Antibacterial screening

In cell-free supernatants without neutralization, 19 out of 47 isolates showed antibacterial activity against the three test pathogens, *B. cereus*, *B. subtilis*, and *E. coli*. Using a supernatant neutralized with 1 N NaOH, we further examined the antibacterial activity of these 19 isolates. Six of the 19 isolates (CW3, CW7, CW14, CW25, CW34, and CW40) showed antibacterial activity against each of the three test organisms. Following an enzyme treatment with protease (20 mg/mL), the neutralized cell-free supernatants of these six isolates were examined for bacteriocin activity against the three test pathogens. Only one sample (CW40) had the zone of inhibition disappear, suggesting the isolate’s bacteriocin activity (Supplementary Figure S1). Table 1 displays isolate CW40's antibacterial activity.

**Table 1:** Antibacterial activity of *Lactobacillus rhamnosus* CW40 without and with neutralization, after enzyme treatment (protease), and purified bacteriocin against indicator organisms.

Test organism	Diameter of inhibition zone (cm)			
	Supernatant without NaOH	Supernatant with NaOH	Neutralized supernatant with protease	Purified bacteriocin
*E. coli*	2.06 ± 0.04^a^	1.73 ± 0.04^a^	NZ	1.89 ± 0.02^a^
*B. cereus*	1.67 ± 0.04^b^	1.43 ± 0.04^b^	NZ	1.56 ± 0.04^a^^,^^b^
*B. subtilis*	1.46 ± 0.04^a^^,^^c^	1.33 ± 0.04^c^	NZ	1.46 ± 0.05^c^

NZ, no zone of inhibition.

Data are presented as means of three replicates ± SD. The mean value followed by the same letter in the column of each treatment is not significantly different at *p* = 0.05 by the Tukey–Kramer HSD test.

### 3.3 Characterization of the isolate

Isolate CW40 was characterized morphologically. It was found that isolate CW40 was rod-shaped and Gram-positive. Isolate CW40 had a white appearance, convex elevation, an entire margin, and pinpointed colonies. Biochemical testing and 16S rRNA partial gene sequencing were used to further characterize isolate CW40. The isolate's catalase activity was found to be negative. On BCP-MRS media, isolate CW40 displayed colonies with a yellow color and hydrolyzed esculin. Isolate CW40 was unable to reduce nitrate and hydrolyze gelatin. It showed a negative result for arginine hydrolysis and HCN production and varied responses for the carbohydrate fermentation reaction. A positive fermentation reaction was observed for mannose, trehalose, and galactose, while a negative reaction was observed for rhamnose and maltose.

**Figure 1:**
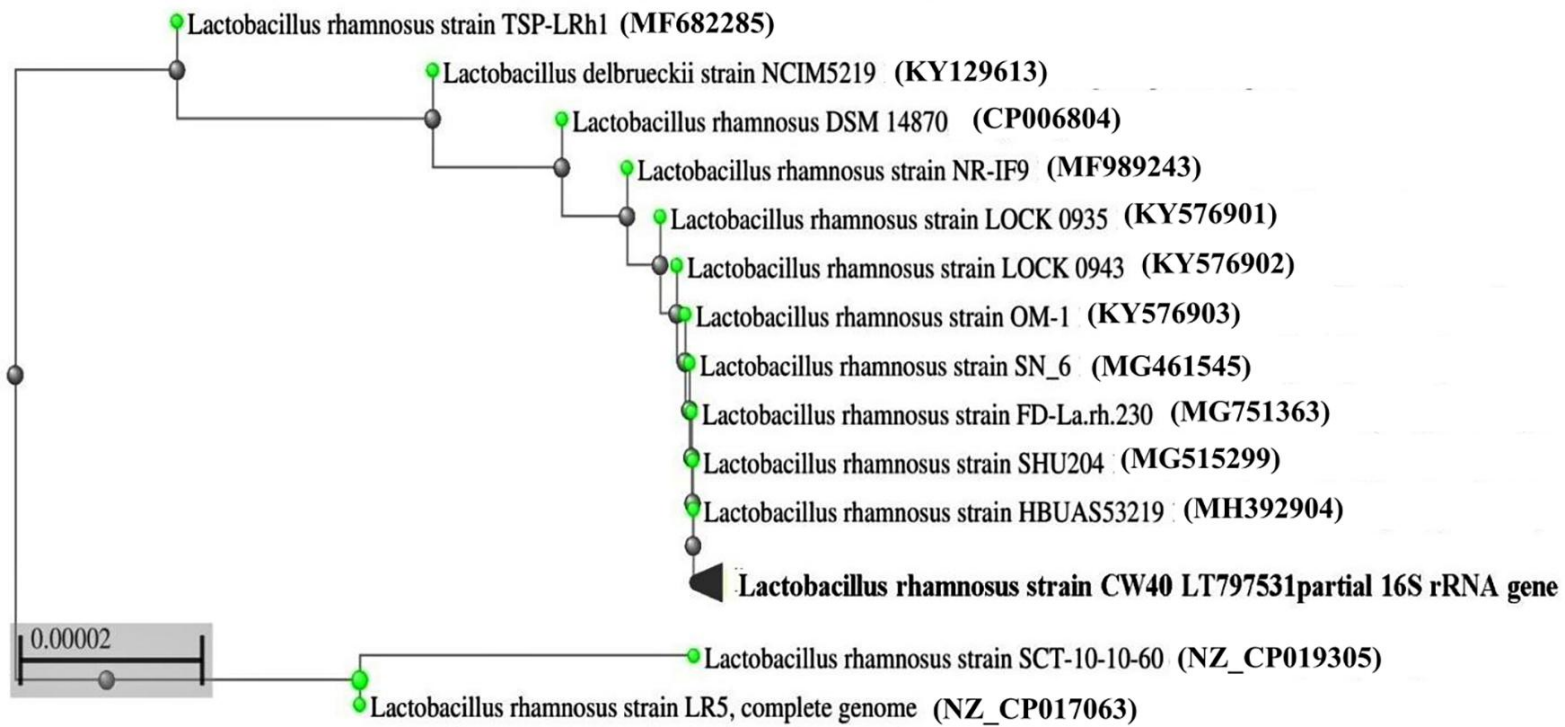
Phylogenetic tree derived from 16S rRNA gene sequences of *L. rhamnosus* CW40 and related type strains. The branch length is proportional to the number of substitutions per site.

Pospeich and Neumann's method was used to extract the isolate's genomic DNA, which was then amplified by PCR using semi-universal primers (Lb1 and Lb2). The 200 bp product that was obtained from isolate CW40 was sequenced. After BLAST analysis, the sequenced data were uploaded to the EMBL gene database with accession number LT797531. Figure 1 shows the phylogenetic relationships between isolate *L. rhamnosus* CW40 and other known *L. rhamnosus* sequences, based on a neighbor-joining tree constructed using the NCBI-BLAST neighbor-joining method.

### 3.4 Production of bacteriocin for determining bacteriocin activity and selection of the indicator organism

The dilution method reported by Daba et al. (20) was used to produce bacteriocin to measure bacteriocin activity. The *Lactobacillus* isolate was grown in 10 mL of MRS broth with 10% inoculum and then incubated at 37°C. After growth was observed, centrifugation was performed to remove the pellet, and the supernatant was filter sterilized and neutralized. The arbitrary unit per milliliter (AU/mL) is used to express bacteriocin activity.

The indicator organism was selected by determining the bacteriocin titer of the cell-free supernatant neutralized with 1 N NaOH of *Lactobacillus rhamnosus* CW40 isolate against *B. cereus*, *B. subtilis*, and *E. coli*. Maximum bacteriocin activity at 4,098 AU/mL was observed against *E. coli*. Based on maximum sensitivity against *Lactobacillus* isolate, *E. coli* was found to be the most effective test organism and was further used as a test organism for studying the effect of pH, temperature, and salt on bacteriocin production.

### 3.5 Production, purification, and molecular weight determination of bacteriocin

**Figure 2:**
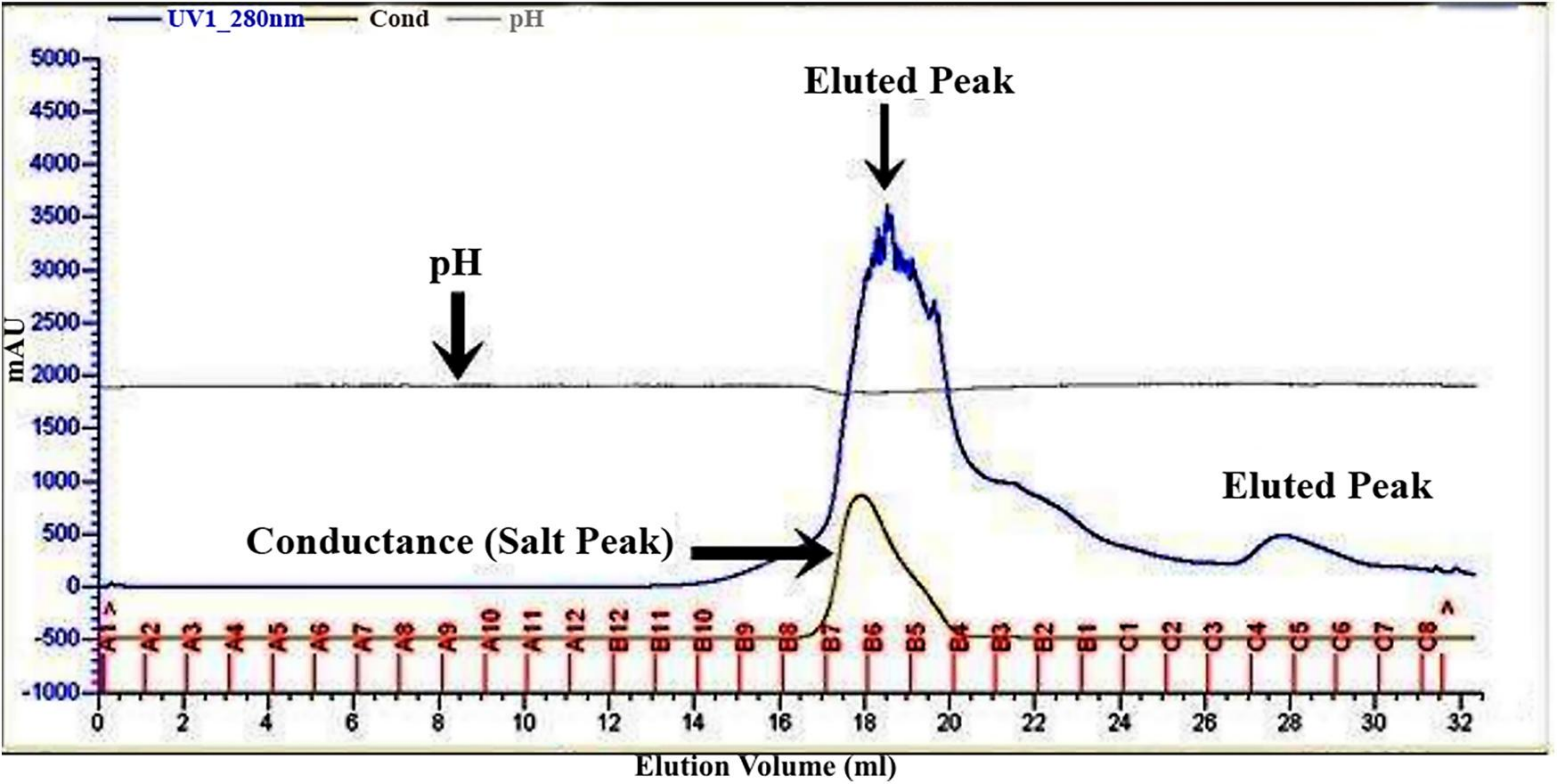
Gel filtration chromatogram of bacteriocin protein by *L. rhamnosus* CW40. Fractions from the peak (B7, B6, B5, B4, B3, and C5) show antimicrobial activity.

The bacteriocin for *L. rhamnosus* CW40 was produced and purified, and its molecular weight was determined. In gel filtration chromatography, peaks were achieved, and respective fractions were collected. Fractions from the peak (B7, B6, B5, B4, B3, and C5 for *L. rhamnosus* CW40) were checked for inhibition against *E. coli*. All the fractions collected showed positive inhibition and were pooled and lyophilized for molecular weight determination. The gel filtration chromatogram of *L. rhamnosus* CW40 is shown in Figure 2. Compared with the crude bacteriocin, the purified bacteriocin had improved antibacterial potency by 9% as compared with the crude bacteriocin (Table 1).

**Figure 3:**
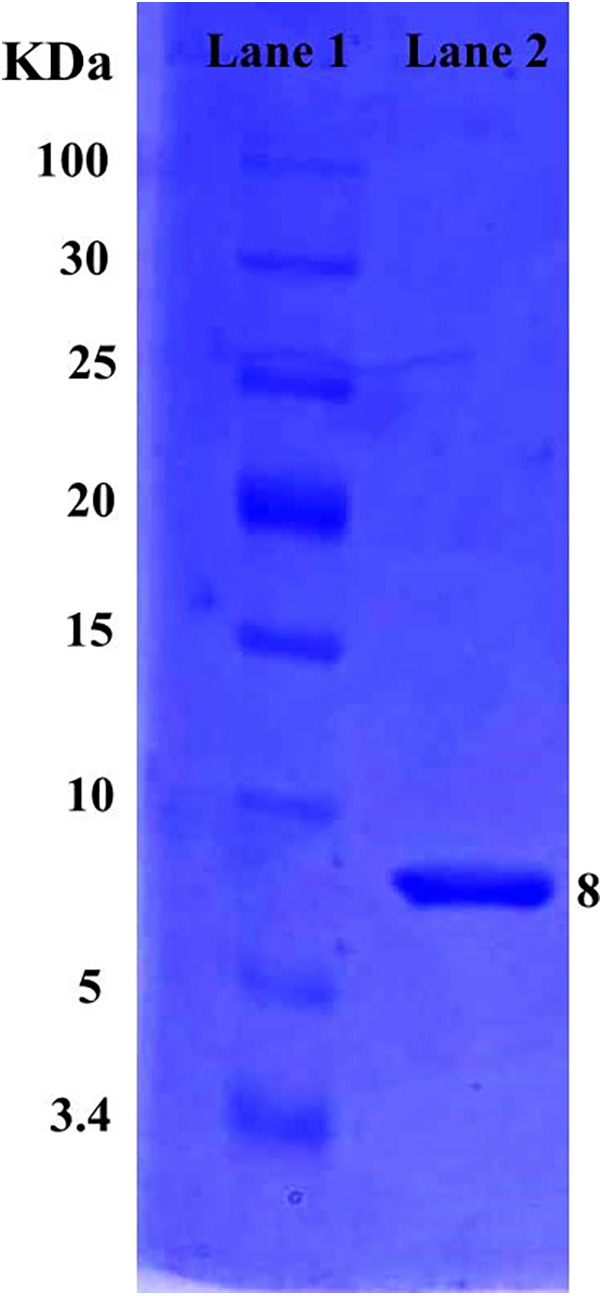
SDS–PAGE gel of *L. rhamnosus* CW40 purified protein sample. Lane 1, low-range protein ladder. Lane 2, purified bacteriocin produced from *L. rhamnosus* CW40.

The molecular weight of a purified protein sample of *L. rhamnosus* CW40 was determined. When *L. rhamnosus* CW40 was stained with Coomassie Brilliant Blue, single-protein bands were observed, confirming the purity of the protein produced after purification. The molecular weight of purified proteins of *L. rhamnosus* CW40 is approximately 8 kDa (Figure 3).

### 3.6 Factors affecting the production of bacteriocin

**Figure 4:**
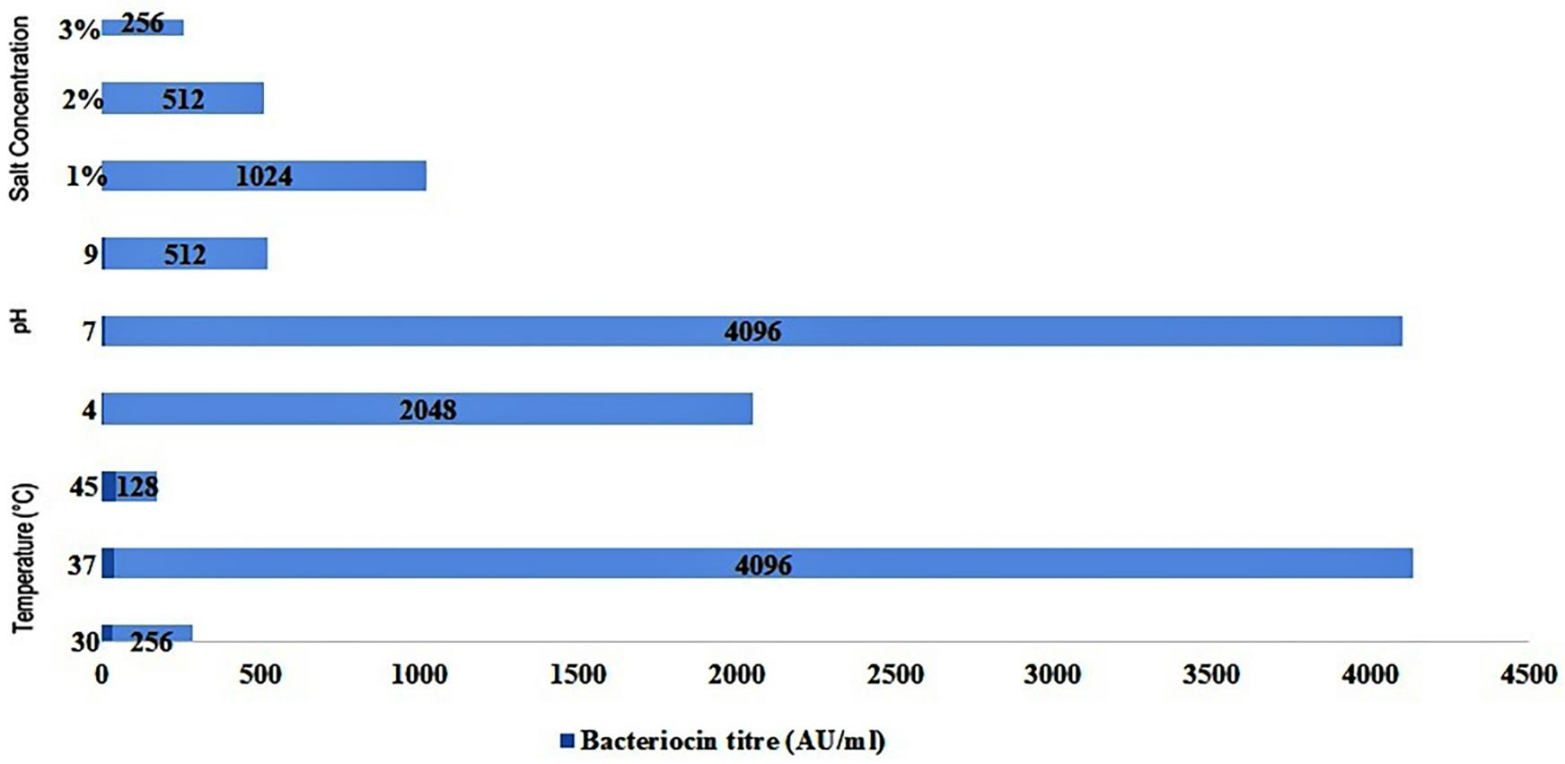
Effect of different incubation temperatures, initial pH, and NaCl concentrations in MRS medium on bacteriocin production by *L. rhamnosus* CW40 against *E. coli*.

The effect of various factors such as pH, temperature, and salt concentration on bacteriocin production by *Lactobacillus rhamnosus* CW40 was studied. In each setup, the effect of incubation temperature, initial pH, and NaCl concentration was determined, and bacteriocin titer in terms of activity unit (AU/mL) was measured after 16 h of incubation (Figure 4). For each experiment, two replicates (microtiter plates) were prepared.

Three different incubation temperatures, i.e., 30°C, 37°C, and 45°C, were used in the study, and bacteriocin titer in terms of AU/mL was determined. The bacteriocin titer produced by the *Lactobacillus* isolate against *E. coli* (indicator organism) varied from 128 to 4,096 AU/mL. At 30°C, a bacteriocin titer of 256 AU/mL was observed for *L. rhamnosus* CW40. At a 37°C incubation temperature, a higher bacteriocin titer than at 30°C was observed in the *Lactobacillus* isolate under study. The highest bacteriocin titer was 4,096 AU/mL for *L. rhamnosus* CW40. At a 45°C incubation temperature, a sharp decline in bacteriocin titer of 128 AU/mL was observed.

Three different initial pH values, i.e., 4, 7, and 9, were maintained, and bacteriocin production in terms of bacteriocin titer was determined. At pH 4, the bacteriocin titer of 2,048 AU/mL was observed for *L. rhamnosus* CW40. At pH 7, a higher bacteriocin titer of 4,096 AU/mL was observed than at acidic pH. At an initial pH of 9, a sharp decline was observed, and the bacteriocin titer shrank down to 512 AU/mL.

In the last setup, three different salt (NaCl) concentrations were maintained, and bacteriocin production in terms of bacteriocin titer was determined. At 1% NaCl concentration, the bacteriocin titer observed was 1,024 AU/mL. At 2% and 3% NaCl salt concentrations, the bacteriocin titer seemed to decline as the salt concentration increased. At 2% NaCl concentration, a bacteriocin titer of 512 AU/mL was observed. At a 3% NaCl concentration, a bacteriocin titer of 256 AU/mL was observed.

### 3.7 Bile tolerance

*Lactobacillus rhamnosus* CW40 showed variable levels of growth in MRS agar supplemented with different ox gall concentrations (0.1%, 0.2%, 0.3%, 0.4%, and 0.5%). After 24 h of incubation, the isolate demonstrated satisfactory growth and was able to withstand a 0.1% ox gall concentration. The isolate was further able to tolerate 0.2% and 0.3% of ox gall concentrations, although it demonstrated relatively reduced growth. Even after 48 h of incubation, no growth was seen, and the isolate was unable to tolerate both 0.4% and 0.5% of ox gall concentrations.

### 3.8 Antibiotic resistance pattern

A total of 12 distinct antibiotics were used to evaluate the antibiotic resistance pattern of *L. rhamnosus* CW40 (Supplementary Figure S2). The isolate was found to be sensitive to six antibiotics (tetracycline, streptomycin, penicillin, rifampicin, amikacin, and gentamycin) and resistant to six antibiotics (ampicillin, kanamycin, cefixime, polymyxin B, trimethoprim, and vancomycin).

## 4 Discussion

The current study focuses on identifying, characterizing, and assessing possible probiotic microbes from cow milk. The impact of various factors, including pH, temperature, and NaCl content, on bacteriocin production was investigated. Protein production, purification, and molecular weight were also determined. Lactobacilli prefer habitats with high carbohydrates, including raw milk, making cow milk a potential source of *Lactobacillus* isolation (25–27). The diverse microbial ecosystem present in raw cow milk offers a broad spectrum of LAB species, increasing the potential for discovering novel bacteriocinogenic strains with significant antimicrobial properties. Upadhyay and Joshi (28) isolated and characterized *L. rhamnosus* CW48 from raw cow milk with probiotic potential.

High inhibition was observed when the un-neutralized cell-free supernatant of all the *Lactobacillus* isolates was tested against indicator organisms. According to Rattanachaikunsopon and Phumkhachorn (29), lactic acid bacteria produce lactic acid and use this acid to inhibit active transport, interfere with the potential of the cell membrane, lower the pH of the cell, and interfere with various metabolic processes. The reason and mode of inhibition of the *Lactobacillus* isolate when un-neutralized cell-free supernatant is utilized against indicator bacteria can be explained. Bacteriocins currently serve as promising natural antimicrobials in food preservation and healthcare, with future potential as targeted, eco-friendly alternatives to conventional antibiotics in combating multidrug-resistant pathogens (Supplementary Figure S3). Bacteriocins' antagonistic activity against foodborne pathogens, such as bacteria and viruses that cause food spoilage, has sparked a lot of interest in their application in food preservation (30). Bacteriocins produced by Gram-positive bacteria generally have antibacterial activity against Gram-positive bacteria, whereas antagonistic behavior against Gram-negative organisms is a relatively rare occurrence, according to reports on *Lactobacillus plantarum* (31) and *Lactobacillus bulgaricus* BB18 (32). In the present investigation, *Lactobacillus rhamnosus* CW40 showed inhibitory spectrum against *E.coli* and *Bacillus* species. A novel LAB, *L. rhamnosus* CJNU 0519, was isolated by Jeong and Moon. It produced the bacteriocin rhamnocin 519 and had a restricted antibacterial spectrum that included *S. aureus* and *L. monocytogenes*. The substantial inhibition of *L. monocytogenes* growth by rhamnocin 519 highlighted the potential of *L. rhamnosus* CJNU 0519 in managing foodborne diseases. According to Alakomi et al. (33), lactobacilli generate organic acids, such as lactic acids, which can permeabilize the bacteria' outer membrane. The process makes it possible for other antimicrobial metabolites to enter the cell and makes pathogens more vulnerable to antimicrobial substances. Because the crude cell-free supernatant is neutralized, lactic acid is absent, which can impact the overall inhibition against indicator organisms. This could be the likely cause of the lower inhibition observed when indicator organisms were tested using neutralized cell-free supernatant. Additionally, this substance lost its antibacterial properties after being treated with a protease enzyme. These findings demonstrate the proteinaceous character of the antibacterial substance produced by LAB. These results agree with those for *L. plantarum* C010 that were previously published (34).

The bacteriocins produced by LAB are produced and purified using various techniques. One of the most frequently used methods for isolation, concentration, and purification of bacteriocin involves salting out (salt precipitation) from culture supernatant, followed by gel filtration chromatography, reverse phase-high performance liquid chromatography (RP-HPLC), etc. Further separation is based on charge, size, or hydrophobic interactions (35, 36). Lv et al. (37) examined the purification, characterization, and mode of action of the bacteriocin Plantaricin JY22, which is produced by *Lactobacillus plantarum*, using the gel filtration approach*.* The method used for protein purification in the current study is consistent with the findings stated above. The present study found that the molecular weight of the bacteriocin from the *Lactobacillus* isolate is 8 kDa*.* As per the classification of bacteriocin by (38), this bacteriocin may fall into class II of bacteriocins. According to Todorov and Dicks (39), who purified 8 kDa bacteriocin from *L. rhamnosus* ST462BZ in skim milk, the outcome of the present work is consistent with their findings. Low molecular mass ionophoric peptides (bacteriocins), such as rhamnosin, can be produced by proliferating probiotic cultures and inhibit various bacteria *in vitro*, including Gram-positive human and foodborne bacterial pathogens (40, 41). Zhao et al. (42) separated and purified the bacteriocin-zrx01 from *Lactobacillus rhamnosus* zrx01 and showed that the bacteriocin-zrx01 might have potential as a natural food preservative. Dimitrijević et al. (40) isolated and identified a low molecular mass (6.4 kDa) bacteriocin, rhamnosin A, produced by *Lactobacillus rhamnosus* strain 68, which showed antibacterial activity and inhibited *Micrococcus lysodeikticus* ATCC 4698. Bacteriocin *XN2* separated from *Lacticaseibacillus rhamnosus* XN2, isolated from yak yoghurt, demonstrated antibacterial activity against *Bacillus subtilis*, *B. cereus*, *Micrococcus luteus*, *Brochothrix thermosphacta*, *Clostridium butyricum*, *S. aureus*, *Listeria innocua* CICC 10416, *L. monocytogenes*, and *Escherichia coli*. The antibacterial activity was estimated to be 3,200 AU/mL after 30 h of cultivation (43). Lou et al. (44) explored the probiotic characteristics of *Lacticaseibacillus rhamnosus* SN21-1 and showed antimicrobial activity against pathogens, including *S. Typhimurium*.

*L. rhamnosus* CW40 showed a significantly higher bacteriocin titer in MRS medium (pH 6.8) at 37°C after 16 h of incubation against *E. coli.* Yang et al. (45) reported that *Lactobacillus* isolates produce high bacteriocin titer in an MRS medium with pH 6.2 at 37°C for 16 h of incubation. Since the isolate produced the most bacteriocin at 37°C, the current study's findings were consistent with those of the previously described investigations. Using *L. acidophilus* LA-5, Amiri et al. (46) found that the optimal temperature for bacteriocin synthesis was 38.71°C, which is comparable to the temperature found in our investigation. *L. rhamnosus* CW40 showed significantly higher bacteriocin titer in MRS medium with initial pH adjusted to 7 at 37°C after 16 h of incubation against *E. coli*. The production of bacteriocin is significantly influenced by the medium's initial pH (47) and proteolytic degradation of bacteriocin (48). The addition of salt to the MRS medium negatively impacted the bacteriocin production in all the *Lactobacillus* strains. *Lactobacillus* isolate in the present study showed decreased bacteriocin titer in all concentrations of NaCl (1%, 2%, and 3%) supplemented in an MRS medium with an initial pH of 7 at 37°C after 16 h of incubation against *E. coli*. The impact of increasing the NaCl concentration on *Lactobacillus* spp. growth and bacteriocin production was examined by Rahman (49). He found that growth and bacteriocin production were adversely affected as the salt concentration increased from 1% to 10%. Given that the addition of salt hindered the formation of bacteriocin, the current study's conclusions are entirely consistent with those of the aforementioned investigations.

One of the most important traits of probiotic organisms, according to Mourad and Eddine (50), is their capacity to endure the impacts of bile salts in the gastrointestinal tract, enabling them to live and help the host. The usual amount of bile salt in the intestines is approximately 0.3% (50). *L. rhamnosus* CW40 showed tolerance to bile salt up to 0.3% ox gall concentration. When bile salt was added to the growth medium at a higher concentration, no growth was seen. According to Gunn (51), relatively high bile salt concentration increases the permeability of the lipid and fatty acid-based bacterial cell membrane. As a result, organisms' tolerance to bile salts is reduced. This may explain why the *Lactobacillus* isolate has low tolerance to higher concentrations of ox gall.

Antibiotic resistance to a wide range of antibiotics is another crucial probiotic characteristic that allows lactobacilli to survive in the gut and benefit their host. *Lactobacillus* species show sensitivity to inhibitors of cell wall synthesis, such as penicillin. These findings support the sensitivity of *L. rhamnosus* CW40 to penicillin. This result is in agreement with the studies conducted by Belletti et al. (52) and Khandelwal et al. (53).

The present study lacks structural characterization of the bacteriocin and its detailed mechanism of action. Additionally, the safety profile and potential cytotoxicity of the bacteriocin were not assessed. So, the future research should focus on elucidating the precise molecular structure and mode of action of the bacteriocin produced by *Lactobacillus rhamnosus* CW40. Investigating the genetic regulation of bacteriocin production and exploring synergistic effects with conventional antibiotics could further enhance its potential for clinical and industrial applications.

## 5 Conclusions

Both the incidence of antibiotic resistance in bacteria and an increasing thirst for products with fewer chemicals are driving the need to explore new alternatives to prevent the overuse of therapeutic antibiotics. *Lactobacillus rhamnosus* CW40 possesses antibacterial activity due to bacteriocin production, bile tolerance, and antibiotic resistance against various antibiotics. These properties form the basis of potent probiotic bacteria; further advanced studies can be conducted, and potent probiotic lactobacilli can be developed. This bacterial isolate has a considerable impact as a starter culture for fermented food products and can have positive benefits when used as a probiotic supplement. Furthermore, this bacterial strain has potential uses in food preservation and as an antibiotic substitute because of its natural origin and safety profile.

## Data Availability

The datasets presented in this study can be found in online repositories. The names of the repository/repositories and accession number(s) can be found in the article/Supplementary Material.
