# Supplementary material for: Optimization of bacteriocin production by Lactobacillus rhamnosus CW40: exploring its therapeutic and antibacterial scope
Source: Front Med Technol. 2025 Sep 12;7:1663924. doi: 10.3389/fmedt.2025.1663924 (PMC12463916; doi:10.3389/fmedt.2025.1663924)
Supplement: Supplementary file 1 [file Table1.docx]

Supplementary Material

# Supplementary Figures

| **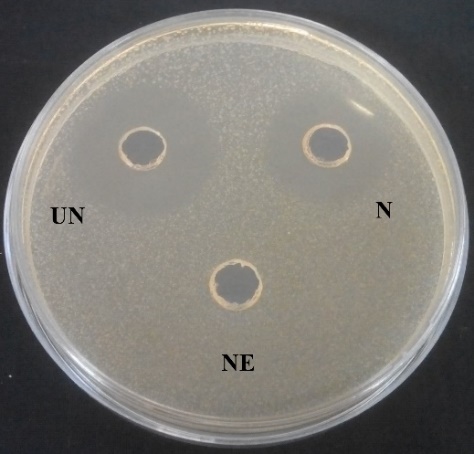** | **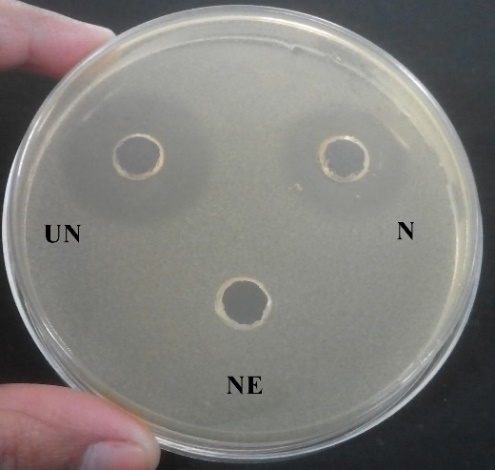** | **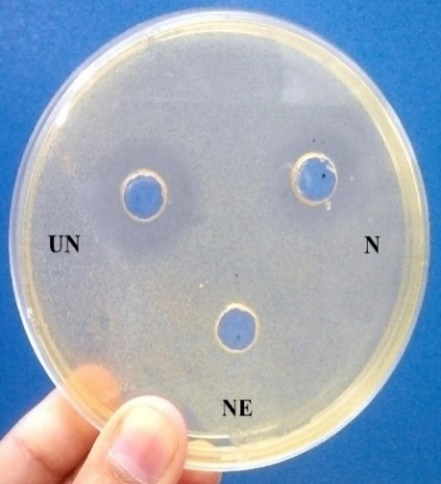** |
| --- | --- | --- |
| **Supplementary Figure 1.** Antibacterial activity of isolates CW on nutrient agar medium at 37˚C after 24h of incubation period against *E. coli* (UN = Un-neutralized crude cell free supernatant, N = Neutralized cell free supernatant and NE = Enzyme treated neutralized cell free supernatant). | | |

| **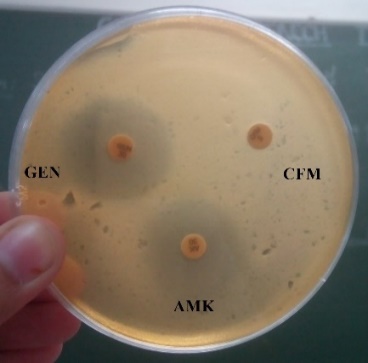** | **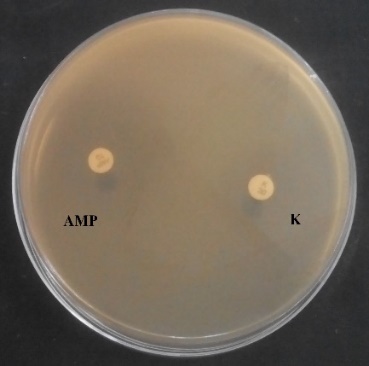** | **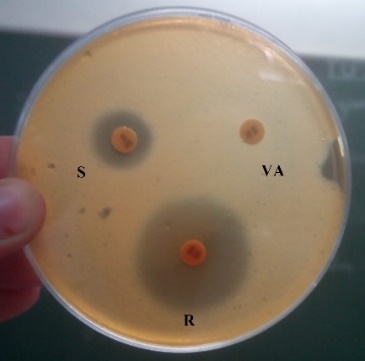** |
| --- | --- | --- |
| **Supplementary Figure 2.** Antibiotic resistance pattern of lactobacilli isolate CW 40 against different antibiotics on MRS agar at 37˚C after 24h of incubation period using disc diffusion method  GEN=Gentamicin, CFM= Cefixime, AMK= Amikacin, AMP= Ampicillin, K= Kanamycin, S= Streptomycin, VA= Vancomycin, R= Rifampicin | | |


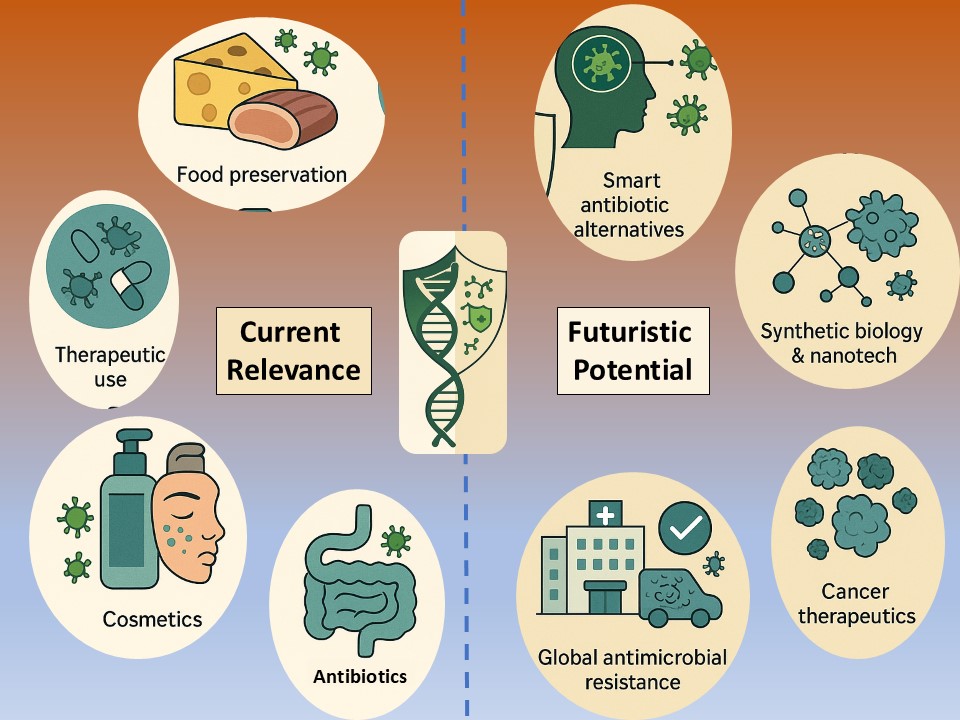


**Supplementary Figure 3.** Significance of bacteriocins: current relevance and futuristic potential
